# Harnessing telehealth for multimorbidity management in rural and remote areas: A scoping review of interventions, outcomes, and implementation dynamics

**DOI:** 10.1177/26335565251344433

**Published:** 2025-06-11

**Authors:** Sage M. C. Ishimwe, Delia Hendrie, Timothy A. Carey, Jacquita S. Affandi, Ninh Thi Ha, Sue Critchley, Amna Mushtaq, Sayyida Anees, Harley Sitou, Chak Seng Song, Brian Leong, Anneli Robbshaw, Christopher M. Reid, Dan Xu

**Affiliations:** 1 Curtin School of Population Health, 1649 Curtin University, Bentley, WA, Australia; 2 Centre for Health Equity in Regional and Remote Communities, 6939 CQUniversity Australia, Rockhampton, QLD, Australia; 3 Royal Australian College of General Practitioners, East Melbourne, VIC, Australia; 4 Curtin Medical School, 1649 Curtin University, Bentley, WA, Australia; 5 Faculty of Medicine, 58816 Macau University of Science and Technology, Macau, SAR, China; 6 School of Public Health and Preventive Medicine, Monash University, Melbourne, VIC, Australia; 7 The First Affiliated Hospital, Sun Yat-Sen University, Guangzhou, China

**Keywords:** multimorbidity, telemedicine, rural health, healthcare, chronic disease, chronic disease management, implementation

## Abstract

**Background:**

Multimorbidity, the coexistence of two or more chronic diseases, affects 37% of adults globally, especially in rural areas with limited healthcare access. This burden leads to poorer health outcomes. Telehealth offers a solution by improving access to care. This scoping review explored the use of telehealth for managing multimorbidity in rural and remote areas.

**Methods:**

A protocol was registered on Open Science Framework. Four databases were searched for peer-reviewed articles published in English from 2010 to 2024, focusing on telehealth interventions for multimorbidity in rural and remote areas.

**Results:**

Out of 9,090 screened records, 15 articles were included in the review. Eight articles reported health outcomes (including five randomised controlled trials), while seven identified facilitators and barriers. Telehealth interventions were categorised as synchronous (5/15) and asynchronous (10/15), and they predominantly targeted physical health conditions (73%). Mixed effects on physiological outcomes were noted, with some studies reporting reductions in blood pressure and glycated haemoglobin. Mental health outcomes generally showed significant reductions in depression and anxiety. Facilitators included telehealth infrastructure, stakeholder engagement, and digital literacy, while barriers reflected the opposite.

**Conclusion:**

This review highlights that telehealth interventions can be cost-effective and improve access and health outcomes in rural and remote areas. However, the variability in findings emphasises the need for standardised implementation and further research to ascertain reliability. Future studies should explore strategies to address barriers and optimise telehealth interventions for managing multimorbidity in these settings.

## Background

Multimorbidity, the coexistence of two or more chronic conditions within an individual,^1^ poses a significant global public health challenge, particularly in rural and remote areas. A recent systematic review and meta-analysis estimated that approximately 37% of the global adult population is affected by multimorbidity.^2^ This condition is particularly prevalent among older adults, individuals with lower socioeconomic status, and those residing in rural or underserved regions, where access to healthcare services is often limited.^3^

The implications of multimorbidity extend far beyond the individual, contributing to broader public health concerns. It is associated with increased mortality rates, reduced quality of life, and higher healthcare utilisation, including more frequent hospitalisations, longer hospital stays, and long-term, complex treatment regimens.^4^ The complexity of managing multiple chronic conditions is compounded by intricate treatment regimens, polypharmacy, and the necessity for coordinated care across various healthcare providers.^5^ Managing multimorbidity thus requires a comprehensive, patient-centred approach that addresses the diverse and often conflicting needs of affected individuals. In rural and remote areas, where healthcare infrastructure is often inadequate and access to multidisciplinary healthcare professionals is limited,^6^ delivering such care becomes particularly challenging. Patients in these regions also face additional barriers related to socioeconomic disparities,^7^ necessitating a reconsideration of traditional healthcare models that have historically focused on single disease management. A shift toward integrated, multifaceted care models is essential to effectively address the complexities of managing multiple chronic conditions.

By leveraging technology, particularly telehealth, healthcare access barriers in rural settings can be effectively addressed. Telehealth includes a range of interventions, such as medical consultations and monitoring through telephone, video calls, or online portals, as well as remote diagnosis.^8^ This well-established approach holds significant potential for improving healthcare accessibility in rural communities.^9^ Research indicates that telehealth can improve care coordination and continuity, offering practical benefits such as increased access to specialists, reduced travel time and costs, and enhanced satisfaction among both patients and clinicians.^10,11^ Despite its potential, telehealth encounters significant challenges in rural populations. These include inadequate broadband infrastructure, varying levels of technological literacy among patients and healthcare providers, and concerns regarding the quality of care delivered remotely.^12^

Previous reviews indicate that digital health interventions can moderately improve multimorbidity management in general populations.^13,14^ However, there is limited evidence focused on adults in rural areas— despite their increased risks due to ageing and persistent rural-urban health disparities, which telehealth interventions are often intended to address.^15^ A search of MEDLINE and JBI Evidence Synthesis found no existing or ongoing reviews addressing this gap. While some reviews have examined telehealth for individual chronic conditions or multimorbidity broadly,^13,14^ none have specifically targeted rural and remote adults.

This scoping review was therefore designed to fill this gap by targeting publications reporting telehealth interventions tailored for multimorbidity management in rural and remote areas. Specifically, it sought to: (i) identify and describe telehealth interventions used in multimorbidity management among adults in rural and remote areas; (ii) identify and describe the reported health outcomes associated with telehealth interventions in managing multimorbidity in rural and remote areas; and (iii) identify the reported facilitators and barriers to implementing telehealth interventions in multimorbidity management in rural and remote areas. By synthesising available evidence, this review will lay the foundation for informed telehealth interventions and more focused studies in this critical and growing area.

## Methods

### Protocol and registration

The scoping review was conducted using the Preferred Reporting Items for Systematic Reviews and Meta-Analyses for Scoping Reviews (PRISMA-ScR) checklist.^16^ We developed a protocol *a priori* based on the PRISMA-ScR checklist and registered it with the Open Science Framework.^17^ The scoping review aimed to identify studies on telehealth interventions implemented in rural and remote areas for managing multimorbidity in adult populations, while highlighting gaps in the existing evidence. Instead of assessing the effectiveness of these interventions, we focused on describing the findings reported in the literature; thus, we did not perform a systematic review or conduct a critical appraisal of the included studies.^18^

### Eligibility criteria

This scoping review included articles involving adult populations (18 years and older) with multimorbidity, specifically those living in rural and remote areas. These areas were defined as regions outside major urban centres, characterised by low population density, significant distances from healthcare facilities, or as defined by the researchers of each study. We included any telehealth intervention addressing multimorbidity (defined as having ≥ 2 chronic conditions), targeting various health outcomes, as well as facilitators and barriers to implementation. Studies focused solely on a single chronic disease, or acute conditions not explicitly aimed at managing multimorbidity were excluded. Studies reporting clinical outcomes were required to include a comparator group (e.g., Usual care) to allow the interpretability of results against standard or alternative care models, as recommended in intervention research.^19^ However, studies on facilitators and barriers to implementation did not require a comparator. The review included original research such as randomised controlled trials (RCTs), cohort, case-control, cross-sectional studies, and quasi-experimental designs. We excluded case reports, case series, editorials, reviews, letters to the editor, corrigenda, retractions and abstracts. Case reports and case series were excluded because they do not contribute empirical data relevant to this review question.

### Information sources and search strategy

The following databases were searched by SMCI (the first author) in collaboration with a faculty librarian: MEDLINE, PsycINFO, Scopus (Elsevier), and ProQuest Central. The searches were restricted to articles published in English from 2010 to 2024 and were completed in December 2024. This timeframe was chosen as it reflects the onset of mainstream telehealth adoption in chronic disease management,^20,21^ a trend that was further accelerated by the COVID-19 pandemic.^22^ Notably, a prior review of telemedicine interventions in chronic disease management, which searched from inception to 2020, identified only one relevant study published before 2010—a 2009 study conducted in an urban setting in South Korea,^13^ which fell outside the scope of this review. A combination of keywords and controlled vocabulary terms (e.g.: Medical Subject Headings (MeSH)) was used to capture each concept of interest: telehealth, multimorbidity, and rural or remote. The search strategy was developed collaboratively by SMCI and the faculty librarian, incorporating input and feedback from the review team members. For details, refer to Supplemental File 1 for the final search strategy. Identified records were then screened following the study selection process outlined below.

### Selection of sources of evidence

A pilot screening process was conducted involving all reviewers on a sample of 25 articles randomly selected by the lead author. The research team then met to discuss any questions and potential changes to the eligibility criteria and overall screening process, which have been documented in the protocol. Screening and study selection were done using Covidence (Veritas Health Innovations). The screening process involved two levels: first titles and abstracts were screened, followed by full text screening. Two reviewers from the team (SMCI, AM, SA, HS, CSS, BL, and DX) independently screened each record in duplicate using the established eligibility criteria at both levels. Disagreements at each level were resolved through consensus discussions during regular virtual meetings, with a third reviewer (TAC, DH, or AR) called upon to adjudicate when consensus could not be reached.

### Data collection, data items and synthesis

Data collection was conducted in Covidence. Before initiating the data collection, reviewers carried out a pilot test with five articles, leading to further clarifications and adjustments to the data collection form and methodology. Once data collection commenced, two reviewers (from SMCI, AM, SA) independently extracted specified data from each article. The extracted data were then compared using the consensus feature in Covidence, and any discrepancies were resolved through discussions between the two reviewers. If necessary, a third reviewer (from JSA, DX, DH, TAC, NTH) was designated to determine the correct data to collect. The data extraction form captured key information, including study characteristics (author, year of publication, country, study design); population details (age, sex, number of participants, targeted chronic conditions); intervention specifics (name and description of the telehealth intervention, duration, rationale, methods of delivery, and achieved outcomes); comparator details, if applicable; as well as reported outcomes (outcome measures, statistical significance), facilitators, barriers, and any other relevant information.

The same two reviewers performed data cleaning and analysis using Microsoft Excel. Numerical analysis and narrative synthesis were used to integrate findings from the included articles. SMCI, with contributions from AM or SA, developed initial narratives and themes aligned with the review’s question.

Telehealth interventions were categorised as synchronous or asynchronous. Synchronous telehealth involves real-time interactions between healthcare providers and patients (e.g., video calls, phone consultations), while asynchronous telehealth allows communication at different times, enabling patients to respond at their convenience (e.g., emails, patient portals).^23,24^ We also grouped targeted chronic conditions (physical, mental, or both), described the reported outcomes, recorded statistical significance, noted the number of articles reporting each outcome, and assessed discrepancies among findings from multiple articles. Before completing the analyses, all the coauthors discussed and reached consensus on the themes. This scoping review presents results from both the numerical analysis and the narrative synthesis of the relevant data identified in the reviewed articles.

### Patient and public involvement

There was no patient or public involvement in this review, as it focused on previously published studies.

## Results

### Search results

**Figure 1.**
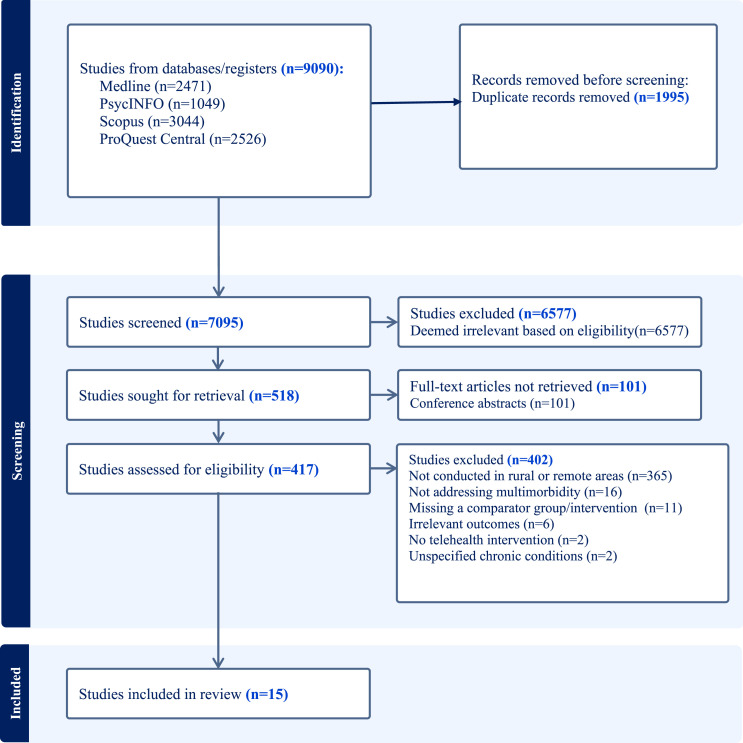
PRISMA flow diagram of the identification of studies through databases.

The databases search yielded 9090 records of which 1995 were duplicates, resulting in 7095 unique records for screening as shown in Figure 1. After reviewing titles and abstracts, 6577 records were excluded. The full text for 101 records were not retrieved as they were conference abstracts. A full-text review of the remaining 417 articles resulted in the exclusion of a further 402 studies based on our inclusion criteria (e.g., studies not conducted in rural or remote areas, lacking relevant outcomes, not addressing multimorbidity, or missing a comparator group/intervention). We retained 15 articles for our review.

### Characteristics of included studies

**Figure 2.**
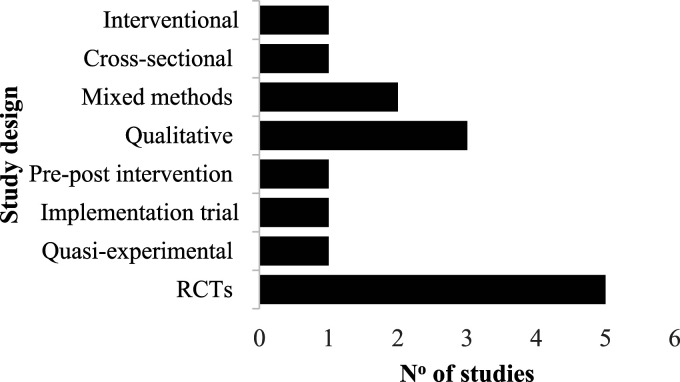
Distribution of study designs among included studies (n = 15).

Table 1 provides a summary of the key characteristics of the included studies; further details are available in Supplemental Table S1. The studies were conducted in eleven countries: Australia (n=2), Canada (n=2), France (n=2), India (n=2), the United States of America (USA; n=1), the United Kingdom (UK; n=1), China (n=1), Taiwan (n=1), Cambodia (n=1), Spain (n=1), and South Korea (n=1). We identified eight studies that documented health outcomes associated with telehealth interventions for managing multimorbidity among adults in rural and remote areas, all of which included control and intervention groups.^25–32^ Of these, five were randomised controlled trials (RCTs),^25,26,30–32^ one employed a quasi-experimental design,^27^ one was an implementation trial,^30^ and one used a pre-post intervention assessment.^28^ Additionally, seven studies explored the facilitators and barriers to implementing telehealth in rural and remote settings.^33–38^ Among these, three were qualitative designs,^34,36,38^ two used mixed methods,^35,39^ one was an interventional study,^37^ and one was a cross-sectional survey,^33^ as depicted in Figure 2.

**Table 1.** Key characteristics of included articles (n = 15).

Author, year country	Telehealth intervention	Targeted chronic conditions		Study design	No. of participants	Outcome measures
		Physical	Mental			
Bohingamu et al., 2019 (Australia)	Remote monitoring and video coaching	DM, COPD	─	RCT	N=177	Hospital admission and LOS; HRQOL; health-related clinical outcomes; anxiety and depression scores; health literacy, and the costs of the intervention and hospitalisations
Chen et al., 2022 (China)	Psychiatrist telephone consultations	HTN	Depression	RCT	N=2,365	Change in depression symptom severity, measured by the HDRS total score and the proportion with controlled HTN.
Lear et al., 2021 (Canada)	Website-based CDM	DM, HF, Ischemic heart disease, CKD, COPD	─	RCT	N=230	All-cause hospitalisations or death at 2 years, hospital length of stay, QoL, self-management, social support, number of participants with ≥ 1 hospitalisation, the time to first hospitalisation, and LOS.
Lan et al., 2022 (Taiwan)	Remote vital sign monitoring	DM, HTN	─	Cross-sectional	N=541	Participants’ satisfaction with the telehealth care system, its usage and usefulness
Kwak et al., 2021 (Korea)	Physician–Nurse telemedicine model	HTN, hyperlipidaemia, DM	─	Quasi-experimental	N=113	Medication adherence using the Korean version of the modified Morisky scale and HRQoL using Korean version of the HRQoL
Guilcher et al., 2013 (Canada)	Tele- CDSMP	Chronic lung disease, heart disease, stroke, arthritis	─	Qualitative	N=44	Participants’ experiences, facilitators and barriers to inform future tele-CDSMP delivery models
Jindal et al., 2018 (India)	mWellcare mobile app	DM, HTN	─	Mixed methods	N=631	Feasibility, acceptability, facilitators and barriers to implementation
Steinman et al., 2020 (Cambodia)	mHealth messaging via MoPoTsyo	DM, HTN	─	Qualitative	N=70	Acceptability, appropriateness, feasibility of mHealth and facilitators and barriers to its implementation
Bean et al., 2021 (USA)	Videoconference-delivered DBT	─	Substance use disorder, depression, anxiety, PTSD	Pre-post intervention	N=69	Changes in mental health symptoms

Author, year country	Telehealth intervention	Targeted chronic conditions		Study design	No. of participants	Outcome measures
		Physical	Mental			
Colomina et al., 2021 (Spain)	eHealth-integrated care	Osteoarthritis	Depression, anxiety	Implementation trial	N=69	SF-12 physical/mental scores, resource use
Tchalla et al., 2023 (France)	Tele-homecare monitoring	COPD, DM, HTN, repetitive fall disorders, CKD, stroke, neurodegenerative diseases, undernutrition	─	RCT	N=534	Incidence of unplanned hospitalisations for decompensation during the 12-month period
Chacornac et al., 2022 (France)	NOMHAD eHealth system	CHF, COPD, DM	─	Interventional	N=23	Users’ experiences and satisfaction
Middlemass et al., 2017 (UK)	Home telemonitoring	COPD, CHF, Ischaemic heart disease	─	Qualitative	N=21	Acceptance, perceptions on the use of the health information technology
Prabhakaran et al., 2019 (India)	mWellcare system with electronic decision support and SMS reminders	DM, HTN	─	RCT	N=552	Changes in BP, HbA1C, fasting glucose, total cholesterol levels, predicted 10-year risk of CVD, depression score, and proportions of participants reporting tobacco and alcohol uses
Schrader et al., 2014 (Australia)	eHealth management program via goACT	DM, HTN, multiple sclerosis, Crohn disease, osteoarthritis, chronic pain, fibromyalgia	PTSD, depression, bipolar disorder	Mixed methods	N=8	Feasibility, acceptability, and barriers to implementation

HDRS: Hamilton Depression Rating Scale; LOS: Length of stay; HRQOL: Health-related quality of life; QoL: Quality of life; CDM: Chronic disease management; CDSMP: Chronic disease self-management program via telehealth; DBT: Dialectical Behaviour Therapy; PTSD: Post-traumatic stress disorder; SF-12: 12-item short-form survey; CHF: Chronic heart failure; HF: Heart failure; COPD: Chronic obstructive pulmonary disease, CKD: Chronic kidney disease; DM: Diabetes Mellitus; HTN: Hypertension; BP: Blood pressure; HbA1C: Glycated haemoglobin; CVD: Cardiovascular disease, UK: United Kingdom.

**Figure 3.**
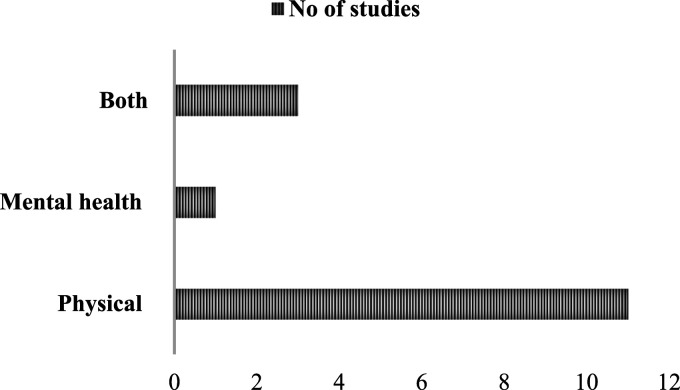
Distribution of target chronic conditions among included studies (n = 15).

Of the interventions, eleven (73%) targeted physical chronic conditions,^25,27,30–38^; three (20%) addressed both physical and mental health conditions,^26,29,39^ and one (7%) targeted mental health conditions alone,^28^ as illustrated in Figure 3. Seven studies included participants with diabetes mellitus and hypertension, either alone,^32,33,35,36^ or with other conditions including hyperlipidaemia, heart failure, chronic obstructive pulmonary disease (COPD), chronic renal failure, recurrent fall disorders, chronic kidney disease (CKD), stroke, neurodegenerative diseases, undernutrition, multiple sclerosis, post-traumatic stress disorder (PTSD), depression, Crohn’s disease, osteoarthritis, anaemia, bipolar disorder, chronic pain, and fibromyalgia.^27,31,39^ Four studies included participants with diabetes mellitus and COPD, either alone,^25^ or with other conditions, including heart diseases, CKD, neurodegenerative diseases, undernutrition, stroke, and repetitive fall disorders.^30,31,37^ Additionally: one study involved participants with hypertension and depression^33^; one with osteoarthritis, depression and anxiety^29^; one with substance use disorder, depression, anxiety, and PTSD^28^; and one with chronic lung disease, heart disease, stroke, and arthritis.^34^

### Telehealth interventions

Table 2 provides a summary of the telehealth interventions used in managing multimorbidity, targeted chronic conditions (physical and mental), and reported outcomes such as health outcomes, enablers, and barriers to implementation. Additional details are available in Supplemental Table S2. All the 15 interventions identified involved at least one element of telehealth. We identified five synchronous,^25–29^ and ten asynchronous telehealth interventions.^30–39^ The duration of the interventions varied: one study lasted 24 months^30^; four studies were 12 months^25,26,31,32^; one study lasted nine months,^38^; one study was six months^29^; two studies were three months^27,37^; two studies were two months^28,35^; one study lasted six weeks,^34^ and three studies did not specify the duration.^33,36,39^

**Table 2.** Overview of the telehealth interventions.

Category	Citation/Intervention	Why? (The rationale for the intervention)	How was the intervention provided?	Duration	Control	Reported outcomes
Synchronous	Bohingamu et al.,Barwon health	Improve outcomes & QoL via home-based monitoring	Remote devices + video coaching from nurses/doctors	12 months	Standard care	↓hospital LOS, ↓costs, ↓ urine microalbuminuria, HbA1C, SOB score, CAT, hospital admissions, ↑HDL, HRQoL, FEV1/FVC, exercise tolerance, health literacy
	Chen et al., eCOACH	Address treatment gaps and social drivers	PCPs + aging worker follow-up; phone psychiatry	12 months	Standard care	↓Depression, ↑ rate of hypertension control
	Kwak et al., Physician-Nurse telemedicine model	Improve medications adherence and HRQoL	Nurse-physician video coordination	3 months	Usual care	↑Medication adherence
	Bean et al., Videoconference-DBT program	Deliver remote care during COVID	Online DBT sessions (90 mins) via Zoom	2 months	In-person care	No significant symptom difference
	Colomina et al., eHealth integrated care	Improve post-surgical chronic care	Mobile app, Fitbit, web profile, case manager	6 months	Previous in-person care	Cost-effective; no change in SF-12
Asynchronous	Lear et al., CDM website	Reduce hospitalisations in multimorbidity	Health goal tracking and nurse follow-up online	24 months	Not reported	↑Self-management, ↓hospital use
	Lan et al., Telemonitoring	Monitor older patients in home/community	Trained volunteers assisted with vitals upload to cloud	Not stated	Not reported	Perceived usefulness, ease of use
	Guilcher et al., Tele-CDSMP	Deliver chronic care education in rural areas	Education via videoconferencing	6 weeks	Not reported	Infrastructure enabled uptake; access barriers
	Jindal et al., mWellcare app	Support nurses to share care tasks	NCD nurse records & updates reviewed by physicians	2 months	In-person DBT-based IOP	Feasible; coordination issues noted

Category	Citation/Intervention	Why? (The rationale for the intervention)	How was the intervention provided?	Duration	Control	Reported outcomes
Asynchronous	Steinman et al., mHealth messaging	To improve patients’ outcomes	Tailored SMS for chronic disease self-care	Not stated	Usual care	↑Engagement; literacy & access limitations
	Tchalla et al., e-COBAHLT remote home monitoring	Prevent rehospitalisation in multimorbidity	Sensor + alert-based telecare post-discharge	12 months	Usual care	↓Readmission & emergency room visits
	Chacornac et al., NOMHAD eHealth	Test technical usability & user experience	Mobile app with vital tracking and color-coded alerts	3 months	Not reported	↑Satisfaction; need for training
	Middlemass et al., Home telemonitoring	Improve QoL & reduce avoidable admissions	Home equipment for symptom tracking	9 months	Usual care	↑Acceptance, ↓hospital admissions
	Prabhakaran et al., mWellcare system	Integrate decision support system at primary care	Decision support system with health records + SMS reminders	12 months	Usual care	No significant difference in SBP, HbA1C, fasting blood glucose, total cholesterol, predicted 10-year risk of CVD, BMI, depression, and tobacco and alcohol use
	Schrader et al., eHealth management program using GoACT platform	Enable collaborative chronic care	CBT + motivational interviewing via mobile app + clinician messaging	Not stated	Not reported	Engagement limited by IT literacy and the burden of illness

↓: Decreased or reduced; ↑: Increased; BMI: Body mass index; CVD: Cardiovascular disease; SBP: Systolic blood pressure; HbA1C: Glycated haemoglobin; NCD: Noncommunicable disease; CHC: Community health center; STAGED: Somatic treatment algorithm for geriatric depression; LOS: Length of stay; COPD: Chronic obstructive pulmonary disease; HDL: High-density lipoprotein; FEV1: Forced expiratory volume; FVC: Forced vital capacity; SOB: Shortness of breath; CAT: COPD assessment test; HRQoL: Health-related quality of life; QoL: Quality of life; PCPs: Primary care providers; DBT: Dialectical Behaviour Therapy; SF-12: 12-item short-form survey; CDM: Chronic disease management; CDSMP: Chronic disease self-management program via telehealth; CBT: Cognitive behavioural therapy.

### Description of the reported outcomes

Table 3 describes the reported outcomes assessing the effects of telehealth interventions on physiological and mental health outcomes, healthcare resource use, cost, medication adherence, self-management, social support, and risk factors. Both statistically significant and nonsignificant changes were reported. Significance and non-significance, in a statistical sense, will be indicated below.

**Table 3.** Overview of the reported outcomes.

Outcome measures	Studies reporting this outcome	Reported outcomes	Significant? (Yes/No)	Any discrepancy? [Yes/No/N/A]	No. of studies with this outcome
Physiological outcomes					
Blood pressure	Prabhakaran et al., 2019	Reduced	No	N/A	1
Proportions of controlled hypertension	Chen et al., 2022	Increased	Yes	N/A	1
Glycated haemoglobin (HbA1C)	Prabhakaran et al., 2019	Reduced	No	No	2
	Bohingamu et al., 2019	Reduced	No		
Fasting glucose	Prabhakaran et al., 2019	Reduced	No	N/A	1
Total cholesterol levels	Prabhakaran et al., 2019	Reduced	No	No	2
	Bohingamu et al., 2019	Increased	No		
COPD assessment Test (CAT)	Bohingamu et al., 2019	Reduced	No	N/A	1
FEV1/FVC	Bohingamu et al., 2019	Increased	No	N/A	1
Shortness of breath score (BORG)	Bohingamu et al., 2019	Reduced	No	N/A	1
Oxygen saturation level (SaO2)	Bohingamu et al., 2019	Increased	No	N/A	1
Urine microalbumin	Bohingamu et al., 2019	Reduced	Yes	N/A	1
LDL/HDL/Triglycerides	Bohingamu et al., 2019	Increased	No	N/A	1
Mental health outcomes					
Depression symptom severity	Prabhakaran et al., 2019	Reduced	No	Yes	2
	Chen et al., 2022	Reduced	Yes		
Anxiety and depression scores	Bohingamu et al., 2019	Reduced	Yes	N/A	1
Depression, anxiety & stress symptoms	Bean et al., 2021	Reduced	No	N/A	1
Health-related quality of life (HRQOL)	Lear et al., 2021	Improved	No	Yes	3
	Kwak et al., 2021	Improved	No		
	Bohingamu et al., 2019	Improved	Yes		
Changes in health status (SF-12)	Colomina et al., 2021	Improved	No	N/A	1
Health care resources use					
Hospital admissions	Colomina et al., 2021	Reduced	Yes	Yes	4
	Tchalla et al., 2023	Reduced	Yes		
	Bohingamu et al., 2019	Reduced	Yes		
	Lear et al., 2021	Reduced	No		
Hospital length of stay (LOS)	Bohingamu et al., 2019	Reduced	Yes	Yes	2
	Lear et al., 2021	Reduced	No		
All-cause hospitalisations or death	Lear et al., 2021	Reduced	No	N/A	1
Emergency/Primary care visits	Colomina et al., 2021	Reduced	Yes	No	3
	Tchalla et al., 2023	Reduced	Yes		
	Bohingamu et al., 2019	Reduced	Yes		
No. of patients with ≥1 admissions	Lear et al., 2021	Reduced	No	N/A	1
Time to first admission	Lear et al., 2021	Reduced	No	N/A	1
Risk of readmission	Tchalla et al., 2023	Reduced	Yes	N/A	1
Cost					
Cost of hospitalisations	Bohingamu et al., 2019	Reduced	Yes	N/A	1
Cost-effectiveness	Colomina et al., 2021	Reduced	Yes	N/A	1
Medications					
Medications adherence	Kwak et al., 2021	Improved	Yes	N/A	1
Self-management and social support					
Self-management	Lear et al., 2021	Improved	Yes	N/A	1
Social support	Lear et al., 2021	Improved	Yes	N/A	1
Health literacy	Bohingamu et al., 2019	Improved	Yes	N/A	1
Risk factors					
Predicted 10-year risk of CVD	Prabhakaran et al., 2019	Increased	No	N/A	1
Exercise tolerance (6MWT)	Bohingamu et al., 2019	Increased	No	N/A	1
Alcohol use	Prabhakaran et al., 2019	Increased	No	N/A	1
Tobacco use	Prabhakaran et al., 2019	Reduced	No	N/A	1

FEV1: Forced expiratory volume; FVC: Forced vital capacity; CVD: Cardiovascular disease; SF-12: 12-item short form survey; 6MWT: 6-min walked test.

For physiological outcomes, blood pressure was reported as reduced in one study, (nonsignificant).^32^ The proportions of controlled hypertension increased in one study (significant).^26^ Glycated haemoglobin (HbA1C) levels decreased in two studies (nonsignificant).^25,32^ Fasting glucose levels were also reported as reduced in one study (nonsignificant).^32^ Urine microalbumin was reduced in one study (significant).^25^ Regarding cholesterol levels, the results were mixed: one study reported a reduction in total cholesterol levels (nonsignificant).^32^; one study reported an increase in high-density lipoprotein (HDL) cholesterol (nonsignificant) ; and^25^ low-density lipoprotein (LDL) cholesterol and triglycerides levels were reported to have increased in one study (nonsignificant).^25^ Various respiratory assessments [COPD Assessment Test (CAT), forced expiratory volume in the first second (FEV1)/ forced vital capacity (FVC) ratio, Shortness of breath score (BORG), Oxygen saturation (SaO2)] reported in one study, generally indicated improvements (all changes were nonsignificant).^25^

In terms of mental health outcomes, depression symptom severity decreased in two studies (one was significant and one was nonsignificant).^26,32^ Anxiety and depression scores were reduced in one study (significant).^25^ Health-related quality of life (HRQoL) showed improvement across three studies (one was significant and two were nonsignificant).^27,30^ Changes in health status (SF-12) were improved in one study (nonsignificant).^29^

Regarding healthcare resource use, four studies reported reductions in hospital admissions (three were significant and one was nonsignificant).^25,29,31^ Hospital length of stay was reduced in two studies (one was significant^17^ and one was nonsignificant^22^). Emergency care visits decreased across three studies (significant).^25,29,31^ All-cause hospitalisations or death was reported reduced in one study (nonsignificant).^30^

For cost-related outcomes, one study reported a reduction in hospitalisation costs (significant),^25^ while another found that the intervention was cost-effective (significant).^29^

Medications adherence improved in one study (significant).^27^ Self-management and social support were reported to have improved in one study (significant).^30^ Also, one study reported an improved health literacy (significant).^25^

In terms of risk factors, one study reported a reduction in tobacco use (nonsignificant) and increases in the predicted 10-year risk of cardiovascular disease (CVD) and alcohol use (nonsignificant).^32^ Additionally, one study indicated an improvement in exercise tolerance as measured by the 6-Minute Walk Test (6MWT) (nonsignificant).^25^

### Identified facilitators and barriers to implementing telehealth in multimorbidity management in rural and remote areas

The reported facilitators and barriers to implementing telehealth in managing multimorbidity were organised into thematic categories. The facilitators were grouped into five main themes: infrastructure; stakeholders’ engagement-patients, healthcare providers, and communities; intervention usability; digital literacy; and identified need. Notably, many barriers identified in the studies often reflected the opposite of these facilitators, so we have presented them together where applicable.

### Infrastructure

Infrastructure played a crucial role in the implementation of telehealth for managing multimorbidity in rural and remote areas across three studies. In the study by Guilcher et al., the presence of existing telehealth infrastructure was identified as a facilitator, enabling the delivery of tailored health education programs across remote communities in Canada.^26^ Significant barriers, however, were also highlighted, such as the lack of reliable Internet access at home and the necessity for patients to travel long distances to telehealth sites, which impeded participation and engagement. Similarly, Chacornac et al. emphasised the importance of infrastructure by reporting that the successful implementation of the NOMHAD eHealth system for patients’ data entry and monitoring in France relied on the time and resources allocated for devices installation.^37^ The study by Steinman et al. in Cambodia reported that existing mobile technology could potentially be used to integrate mHealth solutions for peer educators (PEs) and patients to improve chronic disease management.^28^ The messages were designed to remind users about medications, laboratory tests, doctors’ appointments for consultations, education on how to incorporate self-management into their daily lives, and support for obstacles to disease management. However, frequent changes in patients’ phone numbers hindered the ability to consistently track and follow the recommended health guidelines.

### Stakeholders’ engagement— patients, healthcare providers, and communities

Stakeholders’ engagement was reported as a facilitator and a barrier in the implementation of telehealth intervention across three studies. Jindal et al. reported the critical role of onsite training and orientation programs for all healthcare team members within community health centers while implementing the mWellcare mobile application for health records and patient management.^27^ Although the intervention aimed to shift some responsibilities from doctors to non-communicable disease (NCD) nurses, there was resistance from healthcare staff in following the recommended workflows. Chacornac et al. underscored the significance of allocating time and attention for the training of participants in the successful implementation of the NOMHAD eHealth system for multimorbidity patients’ data entry and management.^29^ The study also noted an improvement in patient satisfaction and the perceived usefulness of the intervention. Middlemass et al. reported that effective organisational processes and informal support systems significantly enhanced the implementation of home telemonitoring for patients with multiple chronic conditions in the UK.^38^

### Usability of the intervention

The usability of the telehealth intervention played an essential role in their successful implementation, as highlighted in two studies. Lan et al. reported that in Taiwan, a telehealth system for monitoring vital signs was designed to be simple.^33^ As a result, participants expressed high levels of user-friendliness and satisfaction with both the quality of information and the services provided. Trained volunteers, referred to as “health gatekeepers,” helped chronically ill patients use a telehealth information system that allowed them to measure vital signs at home. These readings were uploaded to a hospital cloud platform, which maintained continuous health records, analysed data, and monitored for abnormalities. If any issues were detected, the system alerted patients, their families, and healthcare teams via messages, phone calls, emails, or customer service contacts.^33^ Similarly, Middlemass et al. found that an easy-to-use design for home telemonitoring equipment contributed to patients’ perceived ease of use and, hence, its usefulness for individuals with multiple chronic conditions in the UK.^38^

### Digital literacy

Digital literacy has been identified as both a barrier and a facilitator in the implementation of telehealth interventions aimed at managing chronic conditions, as highlighted by findings from two articles. In Schrader et al.'s study, low levels of digital literacy were identified as a significant barrier, impeding patient engagement with the eHealth management program on the goACT platform in Australia.^31^ The intervention incorporated cognitive behavioural therapy, motivational interviewing, and behavioural psychotherapy. Patients evaluated their self-management skills, while healthcare workers accessed and updated results through the digital platform. This platform automated patient support delivery including action and appointment reminders along with email and SMS communication options to complement scheduled meetings or phone contacts between patients and healthcare workers. Similarly, Steinman et al. reported that low digital literacy levels adversely affected patients’ ability to follow recommended health guidelines in the mHealth messaging intervention for peer educators to improve health outcomes through education.^36^

### Identified need

Identifying the gap that an intervention can address is a key facilitator for the successful implementation of telehealth in multimorbidity management, as highlighted by Steinman et al.^36^ In Cambodia, the mHealth messaging intervention supporting MoPoTsyo—a patient information center that trains individuals with diabetes and hypertension to become peer educators—revealed that these peer educators had previously expressed a need for mHealth solutions in terms of patient reminders and health education to help overcome barriers to effective chronic disease management.^36^

## Discussion

This scoping review investigated telehealth interventions for managing multimorbidity among adults living in rural and remote areas. We identified 15 articles from 11 countries, reflecting the global relevance of telehealth interventions in this context. The studies described various interventions, predominantly targeting physical chronic conditions. Overall, telehealth interventions have been reported to positively impact various health metrics, including reducing hospital admissions and healthcare costs, improving medication adherence, and enhancing self-management and health literacy. Many physiological outcomes, however, lacked statistical significance, suggesting the need for further research. We also identified key facilitators and barriers to implementation, including infrastructure challenges, stakeholder engagement, technology usability, and digital literacy.

Interventions were predominantly asynchronous, likely due to the logistical challenges and variability in internet access in rural and remote areas. While real-time telehealth showed promise in fostering engagement and immediacy in care,^40,41^ non-real time delivery modes offer greater flexibility and support in self-management. The greater flexibility and support aligns with studies that highlighted the importance of accommodating patient needs.^42–44^ Despite the diversity of telehealth approaches, most interventions targeted physical health conditions, particularly diabetes and hypertension, while mental health received less attention. This trend may stem from a broader perception that physical ailments are more urgent or quantifiable. However, given the emerging evidence supporting the effectiveness of telehealth in addressing mental health issues,^45^ there is a need to bolster this research in rural areas.

The review indicated a generally favourable trend in physiological outcomes, with statistically significant improvements in urine microalbuminuria and hypertension control.^25,26^ However, improvements in HbA1C, HDL, SaO2, FEV1/FVC, and shortness of breath scores were not statistically significant,^25^ suggesting that these changes may not be directly attributable to telehealth alone. The intervention aimed to enhance health outcomes and quality of life through individualised, cost-effective home-based telehealth monitoring for patients with diabetes and COPD. Discrepancies in outcomes across studies^25,32^ likely arise from heterogeneity in intervention design, baseline patient characteristics, follow-up duration, and how outcomes were defined and measured. For instance, shorter follow-ups may miss longer-term or delayed changes. Standardising outcome measures and intervention periods could enhance comparability in future studies. Despite the lack of statistical significance in some outcomes, clinical relevance may still exist for individual patients. None of the included studies assessed associations between covariates and intervention outcomes—an important gap that future research should address to better tailor interventions to patient subgroups.

In terms of mental health, telehealth interventions significantly reduced depression and anxiety symptoms, showing potential in managing mental health issues within multimorbidity. Additionally, the reported improvements in HRQoL, with one of three studies achieving statistical significance, indicate a possible contribution of telehealth to improving overall wellbeing of rural and remote populations. Further supporting the value of telehealth, reductions in hospital admissions and emergency care visits illustrate the potential of telehealth to alleviate strain on healthcare systems, particularly in remote settings with limited access to care. These findings may advocate for the integration of telehealth into routine care for adults with multimorbidity. Moreover, the decrease in hospital length of stay suggests that telehealth can contribute to fewer acute care needs.

Cost-related outcomes presented a compelling case for the economic viability of telehealth, with significant reductions in hospitalisation costs and improved cost-effectiveness. These findings reinforce telehealth as a cost-effective strategy, especially in healthcare systems seeking to reduce expenditures while improving care quality. The improvement in medication adherence and self-management signifies that telehealth can empower patients to take a more active role in their healthcare. Enhanced health literacy, as reported in one study,^25^ further supports the notion that telehealth provides valuable educational resources, helping individuals navigate some of their health challenges. This empowerment is crucial in rural areas, where patients often encounter additional barriers to accessing health information.^46^ The findings on risk factors were mixed, but the reported improvements in tobacco use and exercise tolerance are encouraging. In contrast, the rise in predicted 10-year CVD risk and alcohol consumption underscores the complexities of managing lifestyle factors through telehealth. This mirrors previous research showing the limited effectiveness of telehealth in modifying dietary habits.^47^ Therefore, future interventions should focus on comprehensive lifestyle modifications that address these areas.

Telehealth implementation success was shaped by several interconnected factors.^31,33,37^ Reliable internet connectivity emerged as a critical barrier, pointing to the need for sustained infrastructure investment. Stakeholder engagement was key to building trust and fostering uptake. In alignment with findings from other rural health studies, community co-design and outreach were found to enhance acceptance and support the long-term sustainability of locally tailored healthcare models.^48–50^ Usability of the intervention also plays an important role, as user-friendly designs enhance patient satisfaction and engagement, especially when paired with support systems like trained volunteers.^33^ However, digital literacy remains a barrier for individuals with lower technological proficiency, suggesting the need for targeted educational initiatives. Additionally, understanding and addressing specific health needs is vital for designing relevant and effective telehealth solutions.

Contextual factors—including health system readiness, workforce availability, family support, and technological infrastructure—played a significant role in shaping the design and outcomes of interventions. These factors influenced not only the types of technologies adopted but also their feasibility, acceptability, and long-term sustainability. As such, context-sensitive approaches are essential to maximise impact in rural and remote areas. Most interventions in this review were not standalone; they were integrated with elements of standard care, including face-to-face assessments, medication reviews, referrals, and follow-ups.^25–27,30,35^ This hybrid model appears to support continuity and enhance outcomes, suggesting telehealth is most effective as a complement—not a substitute—to in-person care. Future research should explore the optimal balance between remote and traditional care for managing multimorbidity. Although this review focused on rural populations, the findings align with broader evidence on telehealth for chronic disease management, which has shown moderate improvements in disease control, health literacy, and psychosocial outcomes.^13,14^ These consistencies highlight telehealth’s potential while also pointing to the need for rural-specific adaptations. Future research and program development should prioritise community co-design, infrastructure investment, and integration with existing services to improve reach, effectiveness, and equity.

### Strengths and limitations

A key strength of our scoping review is that all included studies featured comparator groups, such as standard care or non-telehealth interventions when reporting clinical outcomes. Additionally, most of the articles employed RCTs, which enhances the methodological rigor and validity of their reported findings. However, the limited number of available articles may restrict the generalisability of our findings and the depth of our analysis. By excluding non-English language literature, we may have overlooked relevant studies published in other languages or journals not indexed in the databases we searched. Furthermore, the variability in study designs and intervention durations may impact the consistency in the reported findings, suggesting a need for more standardised approaches in future research.

## Conclusion

While telehealth shows great promise in managing multimorbidity in rural and remote areas, systematic evaluations of its interventions are limited, resulting in weak supporting evidence that hinders broader adoption. Most telehealth interventions we found were combined with different components of usual care, with specific components varying across studies. Standardising intervention protocols—specifying duration and attainable outcomes—is essential for generating reliable and comparable data. Tailoring interventions to the needs and preferences of patients and healthcare providers will enhance the relevance, sustainability, and effectiveness of the interventions. Future research should involve longer follow-up periods and investigate which healthcare components can be best integrated to avoid inappropriately diverting resources from alternative and non-telehealth approaches. Incorporating realistic evaluation methods will be crucial, given the significant impact of contextual factors on implementation and outcomes. Successful telehealth implementation will require addressing infrastructure challenges, fostering stakeholder engagement and improving usability.

## Supplemental Material

Supplemental Material - Harnessing telehealth for multimorbidity management in rural and remote areas: A scoping review of interventions, outcomes, and implementation dynamics. Supplemental Material for Harnessing telehealth for multimorbidity management in rural and remote areas: A scoping review of interventions, outcomes, and implementation dynamics by Sage M.C. Ishimwe, Delia Hendrie, Timothy A. Carey, Jacquita S. Affandi, Ninh Thi Ha, Sue Critchley, Amna Mushtaq, Sayyida Anees, Harley Sitou, Chak Seng Song, Brian Leong, Anneli Robbshaw, Christopher M. Reid and Dan Xu in Journal of Multimorbidity and Comorbidity

Supplemental Material - Harnessing telehealth for multimorbidity management in rural and remote areas: A scoping review of interventions, outcomes, and implementation dynamics. Supplemental Material for Harnessing telehealth for multimorbidity management in rural and remote areas: A scoping review of interventions, outcomes, and implementation dynamics by Sage M.C. Ishimwe, Delia Hendrie, Timothy A. Carey, Jacquita S. Affandi, Ninh Thi Ha, Sue Critchley, Amna Mushtaq, Sayyida Anees, Harley Sitou, Chak Seng Song, Brian Leong, Anneli Robbshaw, Christopher M. Reid and Dan Xu in Journal of Multimorbidity and Comorbidity

Supplemental Material - Harnessing telehealth for multimorbidity management in rural and remote areas: A scoping review of interventions, outcomes, and implementation dynamics. Supplemental Material for Harnessing telehealth for multimorbidity management in rural and remote areas: A scoping review of interventions, outcomes, and implementation dynamics by Sage M.C. Ishimwe, Delia Hendrie, Timothy A. Carey, Jacquita S. Affandi, Ninh Thi Ha, Sue Critchley, Amna Mushtaq, Sayyida Anees, Harley Sitou, Chak Seng Song, Brian Leong, Anneli Robbshaw, Christopher M. Reid and Dan Xu in Journal of Multimorbidity and Comorbidity

## Consent to participate

There are no human participants in this article and informed consent is not required.

## Data Availability

This scoping review synthesises existing literature and does not involve the collection of primary data. Therefore, there are no original datasets to share. All relevant studies included in this review are cited within the manuscript and can be accessed through the respective journals or databases. *
