# Supplementary material for: Harnessing telehealth for multimorbidity management in rural and remote areas: A scoping review of interventions, outcomes, and implementation dynamics
Source: J Multimorb Comorb. 2025 Jun 11;15:26335565251344433. doi: 10.1177/26335565251344433 (PMC12163256; doi:10.1177/26335565251344433)
Supplement: Supplemental Material - Harnessing telehealth for multimorbidity management in rural and remote areas: A scoping review of interventions, outcomes, and implementation dynamics. [file sj-pdf-1-cob-10.1177_26335565251344433.pdf]

Supplemental Table S1: Detailed characteristics of included articles (n=15)

Author, year of publication, and country of origin	Telehealth intervention (For details, please refer to Table 2)	Targeted chronic conditions		Study design	Population	Number of participants	Outcome measures
		Physical	Mental				
Bohingamu et al., 2019<br>Australia	Barwon Health personalised telehealth remote patient monitoring and videoconferencing health coaching sessions.	Diabetes<br>COPD		RCT with an economic analysis	People with diabetes and/or COPD who were admitted to Barwon Health for any reason between November 2012 and November 2013.	N=177 (Intervention n=89 & Control n=88); Drop out=6 (2 in Intervention & 4 in the Control)	Hospital admission and length of stay (LOS); health-related quality of life (HRQOL); health-related clinical outcomes; anxiety and depression scores; health literacy, and the costs of the intervention and hospitalisations.
Chen et al., 2022<br>China	The Chinese Older Adult Collaborations in Health (COACH) intervention: Telephone consultations with psychiatrists.	Hypertension	Depression	RCT	Patients aged 60 years+ registered in the village's primary care; and have a chart diagnosis of hypertension and clinically significant depressive symptoms.	N=2,365 (Intervention n=1,232 & Control n=1,133); Drop out/Died=152 (85 in Intervention & 67 in the Control)	Change in depression symptom severity, measured by the Hamilton Depression Rating Scale (HDRS) total score and the proportion with controlled hypertension.

Lear et al., 2021<br>Canada	Internet chronic disease management (CDM) via a website for coordinated care.	Diabetes<br>Heart failure<br>Ischemic heart disease<br>CKD<br>COPD		RCT	Participants with chronic conditions aged > 19.	N=230 (Intervention n=117 & Control n=113); Drop out=1 from the Intervention group.	All-cause hospitalisations at 2 years,<br>hospital length of stay, quality of life, self-management, social support, number of participants with at least 1 hospitalisation, the number of participants who experienced a composite outcome of all-cause hospitalisation or death, the time to first hospitalisation, and the number of in-hospital days.
Lan et al., 2022<br>Taiwan	Telehealth care system for monitoring vital signs with data transmitted to a hospital platform.	Hypertension<br>Diabetes		Cross-sectional survey	Patients≥40 years medically diagnosed with diabetes and/or hypertension; using a telehealth care system.	N=541 respondents (4 questionnaires excluded for inconsistency).	Participants' satisfaction with the telehealth care system, its usage and usefulness.
Kwak et al., 2021<br>Korea	Physician-Primary-Healthcare Nurse Telemedicine Model (P-NTM).	Hypertension<br>Hyperlipidaemia<br>Diabetes		Quasi-experimental, non-equivalent-control-group pre-post-test.	Patients with hypertension, hyperlipidaemia, and diabetes who visited primary-healthcare centers or clinics located in 11 remote rural areas.	N=113	Medication adherence using the Korean version of the modified Morisky scale and health-related quality of life (HRQoL) using Korean version of the HRQoL.
Guilcher et al., 2013<br>Canada	Chronic disease self-management program via telehealth (Tele-CDSMP).	Chronic lung disease<br>Heart disease<br>Stroke<br>Arthritis		Qualitative	Patients aged ≥ 45, self-reported physician diagnosis of chronic lung disease, heart disease, stroke, or arthritis.	N=44	Participants' experiences, facilitators and barriers to o inform future tele-CDSMP delivery models.

Jindal et al., 2018 India	mWellcare mobile application for health records and patient management.	Hypertension Diabetes		Mixed methods	Patients diagnosed with hypertension and/or diabetes attending community health centers (CHCs).	N=631	Feasibility, acceptability, facilitators and barriers to implementation.
Steinman et al., 2020 Cambodia	mHealth messaging intervention supporting MoPoTsyo [a patient information center that trains individuals with diabetes and/or hypertension to become peer educators (PEs) for their communities].	Diabetes Hypertension		Qualitative	PEs and people living with diabetes and/or hypertension.	N=70 (59 patients and 11 PEs).	Acceptability, appropriateness, feasibility of mHealth and facilitators and barriers to its implementation.
Bean et al., 2021 USA	Videoconference-delivered Dialectical Behaviour Therapy (DBT) program.		Substance use disorder Depression Anxiety PTSD	Pre-post intervention assessment	Individuals with comorbid mental health, who completed the dual diagnosis IOP at a suburban private practice located in Northeast Ohio from January 2018 through June 2021.	N=69 (Intervention n=20 & Control n=49)	Changes in symptoms of depression, anxiety, and stress following completion of the IOP was compared between the in-person and videoconference groups.

Colomina et al., 2021 Spain	Integrated Care (IC) with an eHealth platform involving real-time data and interactions.	Osteoarthritis	Depression Anxiety	Prospective, pragmatic, two-arm, parallel implementation trial	Home-dwelling patients elected for primary total hip arthroplasty (THA) or total knee arthroplasty (TKA) at the University Hospital of Santa Maria; aged >65 years.	N=69 (Intervention n=39 & Control n=30); Drop out=10 from the Intervention group.	Changes in the 12-item short-form survey (SF-12) health questionnaire's physical and mental domains (baseline vs. discharge); use of health care resources after 6 months (admissions, emergency visits); and cost-effectiveness.
Tchalla et al., 2023 France	e-COBAHLT_The remote home monitoring program using biometric technology to analyse home life and facilitate tele-homecare.	COPD<br>Diabetes<br>Hypertension<br>Repetitive fall disorders Chronic renal failure<br>Stroke<br>Neurodegenerative diseases<br>Undernutrition		RCT	Patients aged ≥ 65, who had been hospitalised in the previous year for the following conditions of interest in this study (See the column of targeted chronic conditions).	N=534 (Intervention n=267 & Control n=267); Died=39 (19 in Intervention & 20 in the Control)	Incidence of unplanned hospitalisations for decompensation during the 12-month period.
Chacornac et al., 2022 France	NOMHAD eHealth system for data entry and monitoring via mobile app.	Chronic heart failure (CHF)<br>COPD<br>Diabetes		Single-arm interventional study	Adults >18 years with at least two chronic diseases among CHF, COPD, and diabetes and with at least one hospitalisation.	N=23	Users' experiences and satisfaction.

Middlemass et al., 2017 United Kingdom	Home telemonitoring for patients with multiple diseases.	COPD<br>CHF<br>Ischaemic heart disease		Nested qualitative study as part of a multicentre international clinical trial	Patients aged > 60 years, with severe COPD and associated CHF or IHD; with a current or prior history of smoking and an exacerbation in the past year requiring hospitalisation and/or antibiotics).	N=21	Acceptance, perceptions on the use of HIT.
Prabhakaran et al., 2019 India	mWellcare system with electronic decision support and SMS reminders.	Hypertension<br>Diabetes		RCT	≥30 years of age, intended to reside in the catchment area of CHCs for 1 year, and had been diagnosed with hypertension or type 2 diabetes mellitus.	N=552 (Intervention n=253 & Control n=299)	Changes in BP, HbA1C, fasting glucose, total cholesterol levels, predicted 10-year risk of CVD, depression score, and proportions of participants reporting tobacco and alcohol uses.

Schrader et al., 2014<br>Australia	eHealth management program using goACT platform to enable communication between patients and clinicians.	Diabetes<br>Hypertension<br>Multiple sclerosis<br>Crohn disease<br>Osteoarthritis<br>Chronic pain<br>Fibromyalgia	PTSD<br>Depression<br>Bipolar disorder	Mixed methods	Patients with chronic physical and psychological comorbidities, aged 49 to 78, recently hospitalised, participated in the eHealth program within the past 2 years and have mobile phones and internet access at home.	N=8	Feasibility, acceptability, and barriers to implementation.
