# Supplementary material for: Harnessing telehealth for multimorbidity management in rural and remote areas: A scoping review of interventions, outcomes, and implementation dynamics
Source: J Multimorb Comorb. 2025 Jun 11;15:26335565251344433. doi: 10.1177/26335565251344433 (PMC12163256; doi:10.1177/26335565251344433)
Supplement: Supplemental Material - Harnessing telehealth for multimorbidity management in rural and remote areas: A scoping review of interventions, outcomes, and implementation dynamics. [file sj-pdf-2-cob-10.1177_26335565251344433.pdf]

Supplemental Table S2: Details of the telehealth interventions

Category	Citation/ Intervention	Why? (The rationale for the intervention)	How was the intervention provided?	Duration of the intervention	Control group/Intervention	Reported outcomes
Synchronous	Bohingamu et al., Barwon Health personalised telehealth remote patient monitoring and videoconferencing health coaching sessions.	To improve health outcomes and quality of life for patients with diabetes and COPD through cost-effective home-based telehealth monitoring.	Participants used peripheral devices (e.g.: sphygmomanometer) and tablets to collect biometric data and complete daily health questionnaires through the TELUS Canada Remote Patient Monitoring platform. Each had a personalised care plan developed by a specialist and a remote monitoring nurse, who monitored vital signs and glucose levels. Nurses were available for support anytime. Participants also received biweekly health coaching sessions via videoconferencing. Continuous communication between nurses and GPs ensured coordinated care, with referrals as necessary, and lead medical consultants oversaw and adjusted monitoring parameters when needed.	12 months	Standard care: Participants were advised to manage their condition as usual, with their community GP, Barwon Health outpatient clinic appointments, and emergency department where appropriate.	Decreased hospital length of stay (LOS), reduced hospital cost, reduced urine microalbuminuria, HbA1C, shortness of breath score, COPD assessment test (CAT), hospital admissions, increased HDL, HRQoL, FEV1/FVC, exercise tolerance, health literacy.
	Chen et al., The Chinese Older Adult Collaborations in Health (COACH) intervention: Telephone consultations with psychiatrists.	To help reinforce treatment and address social factors affecting health, and telephone-based consultation with a psychiatrist.	Primary care providers (PCPs) saw participants at baseline and again at regular intervals, at least monthly, for follow-up depression screening (PHQ-9) and BP checks. - The aging workers (Aws) educated participants about their conditions followed by goal setting and collaborative development of behaviour change strategies through monthly home visits. -The psychiatrist provided initial in-person consultation with the PCP in the	12 months	enhanced care-as-usual (eCAU): Primary care providers (PCPs) were told when their patients screened positive for depression and were provided with copies of antidepressant treatment guidelines adapted from the Duke	Improvement in both depressive symptoms and the rate of hypertension control.

			village clinic and made the initial prescription of antidepressants as indicated. Thereafter, participants met with the PCP and AW monthly by telephone to review cases and make recommendations.		Somatic Treatment Algorithm for Geriatric Depression (STAGED).	
	Kwak et al., Physician-Primary-Healthcare Nurse Telemedicine Model (P-NTM).	To improve medication adherence and health-related quality of life (HRQoL).	Primary healthcare nurses used computers or video communications to send patient information to remote physicians, who then provide prescriptions and treatment recommendations.	3 months	Usual care: Participants were given educational information on chronic disease management and a list of internet-based resources. Aside from outcome assessments at 12 and 24 months, there was no further contact with study personnel, and participants were free to seek any type of care.	Improved medication adherence after participating in P–NTM
	Bean et al., Videoconference-delivered Dialectical Behaviour Therapy (DBT) program.	To transition from in-person treatment delivery to a videoconference-delivered DBT-based IOP for individuals with comorbidmental health and substance use disorder diagnoses inresponse to the pandemic.	Trained group leaders and psychologists always started the intervention by adjusting for the Zoom platform, such as providing email handouts and support for connectivity issues. Each session lasted about 90 minutes, starting with a 30–45-minute review of the previously introduced DBT skill. Patients then discussed their individual diary cards, rating their emotional experiences and the skills they used since the last session. Brief therapy (around 5 minutes) was offered to address any increased stressors. A new DBT skill was introduced toward the end of the session, which	2 months	Not available	No significant differences in symptom reduction were found between the groups.

			included a 15-minute break for participants to engage in various activities.			
	Colomina et al., Integrated Care (IC) with an eHealth platform involving real-time data and interactions.	To assess the effectiveness and cost-effectiveness of implementing a mobile health (mHealth)–enabled IC model for complex chronic patients undergoing primary total hip or knee arthroplasty.	Patients received care through an IC model, which included: (1) a preliminary health assessment using various questionnaires and tests tailored to their chronic diseases and social needs; (2) a self-management app featuring status and performance reports, a virtual coach with customisable feedback, and communication with the care team; (3) a Fitbit Flex 2 activity tracker integrated into the app; (4) a patient profile on a web-based platform accessible to the entire care team, facilitating coordination and communication; and (5) a designated case manager to supervise the process and serve as the primary contact for patients. After an initial 90-day period of integrated care management, patients continued with an additional three months of passive follow-up.	6 months	Previous face-to-face hospital care	No significant difference in the SF-12 physical domain and total SF-12 scores between both groups. Cost-effective (saving approximately $132.96 to $153.66 per patient by reducing unplanned visits and hospitalisations).
**Asynchronous**	Lear et al., Internet chronic disease management (CDM) via a website for coordinated care.	To reduce hospitalisations among patients with multiple chronic diseases.	CDM website was designed by a committee of clinical experts and patient representatives. Each patient received unique login credentials and training for the CDM website. The nurse conducted initial calls to discuss health conditions, co-develop action plans, and set biometric targets. Participants regularly completed symptom reports and biometric data. Alerts were generated for unmet targets, prompting the nurse to contact	24 months	Not available	No significant difference in all-cause hospitalisations. Improved self-management and social support. Fewer hospitalisations.

			participants to provide support, recommend PCP follow-ups, or refer to a hospital if necessary.			
	Lan et al., Telehealth care system for monitoring vital signs with data transmitted to a hospital platform.	To monitor older and chronically ill patients at home and in the community.	Trained volunteers, "health gatekeepers," assisted chronically ill patients in using a telehealth information system. Patients could measure vital signs at home, which were then uploaded to a hospital cloud platform. This platform maintained continuous health records, analysed data, and monitored for abnormalities. If issues were detected, it alerted patients, their families, and healthcare teams by message, phone line, email, or customer service contact so the medical staff could stay informed about patients' status at home.	Not specified	Not available	Facilitators: Making the system simple and easy to use. The technology's perceived ease of use and usefulness helped users find the intervention helpful.
	Guilcher et al., Chronic disease self-management program via telehealth (Tele-CDSMP).	To provide education programs targeted toward effectively managing chronic conditions to people living in rural and remote settings.	Tailored health education courses were delivered across 13 Northern Ontario communities that had existing telehealth infrastructure, using a videoconferencing system.	6 weeks	Not available	Facilitators: Existing telehealth infrastructure, strong program leaders, enough time to be comfortable with technology. Barriers: lack of Internet access home, long travels to telehealth sites.

	Jindal et al., mWellcare mobile application for health records and patient management.	To use the NCD nurse for patient assessment and long-term follow-up using mWellcare system thereby shifting/sharing some of the patient management tasks from doctors.	The mWellcare application was installed on tablets for use by NCD nurses. Nurses logged in to register patients diagnosed with hypertension and/or diabetes. They conducted initial evaluations using the app, generating a Decision Support Recommendation (DSR) printout for the patient to take to their physician. After the physician reviewed the DSR, the patient returned to the NCD nurse to update the DSR in the mWellcare system and receive lifestyle advice based on the physician's recommendations.	2 months	In-person DBT-based IOP	Facilitators: Emphasised onsite training and orientation program to cover all health care team member in each CHC. Barriers: resistance to follow mWellcare recommended patient workflow, due to lack of communication between pharmacist, registration desk.
	Steinman et al., mHealth messaging intervention supporting MoPoTsyo [a patient information center that trains individuals with diabetes and/or hypertension to become peer educators (PEs) for their communities].	To improve MoPoTsyo patients' clinical outcomes.	The research team used findings from interviews and focus group discussions with patients to develop mHealth cell phone messages to improve MoPoTsyo patients' clinical outcomes. Specifically, the messages were created to improve evidence-based chronic disease management via better access to health care (doctor's consultations, laboratory monitoring, medications, and PEs) and self-management (diet, weight management, physical activity, alcohol, and smoking).	Not specified	Usual care: Participants were attended to using usual care and managed from primary care after hospital discharge.	Facilitators: The identified need for mHealth solutions (reminders and educational resources) to help overcome barriers to chronic disease management. Barriers: Frequent changes in cell phone numbers, low literacy, and limited access to medications and health resources affected patients' ability to follow recommended health guidelines.

	Tchalla et al., e-COBAHLT remote home monitoring program.	To prevent rehospitalisations in older patients with two or more chronic diseases returning home after hospitalisation.	Patients received educational sessions about the nurse-led telemonitoring program. Participants were equipped with automation sensors and used a telemonitoring kit featuring e-GEROPASS software—an algorithmic tool that analysed clinical data (blood pressure, heart rate, body temperature, oximetry, blood sugar, weight, and heart activity) and flagged any out-of-range results. Data were transmitted via Bluetooth to a hub, ensuring continuous 24/7 monitoring. Geriatricians communicated feedback and potential therapeutic adjustments to healthcare personnel, who received alerts on their phones to access the software for updates.	12 months	usual care from their general practitioner (GP).	Significantly lowered the risk of rehospitalisation and reduced the number of unscheduled hospitalisations and emergency room admissions.
	Chacornac et al., NOMHAD eHealth system for data entry and monitoring via mobile app.	To evaluate the technical performance and user experience of the NOMHAD eHealth system in patients with multimorbidity.	Consists of the NOMHAD Chronic software platform and the NOMHAD Mobile app, which patients use on an interactive tablet to input vital signs—either manually or through connected devices—and complete questionnaires. This data is transmitted to the software platform via 3G, 4G, or Wi-Fi connections. The system enables remote monitoring of symptoms and vital signs, paired with structured tele-coaching and educational support from call center nurses. After each data transmission, an Overall Risk Indicator (ORI) is automatically calculated and color-coded: green indicates no action is needed, while yellow, orange, and red signal low to high priority, prompting nurses to take necessary tele-	3 months	Not available	Facilitators: Time and attention allocated to the study devices installation and training participants on the eHealth system. Beneficiaries' satisfaction, usefulness of the intervention.

			coaching actions.			
	Middlemass et al., Home telemonitoring for patients with multiple diseases.	To reduce unnecessary hospital admissions and im-prove quality of life for the patient.	Participants were given the equipment in their own home with a clinical alert system. The equipment was designed to measure lung function. The Wrist clinic was given only to those with confirmed CHF. Patients entered daily responses through a computer monitor to a number of symptom questions relating to their illness. Clinical alerts were created in response to changes in the measures being monitored which could indicate deterioration and predict worsening of the condition, so that remedial action could be taken, either by the patients themselves or by the nurse or other health professional.	9 months	Usual care without health information technology (HIT).	Facilitators: Good organisational processes and informal support; Ease of use of the equipment design, Perceived/Proven usefulness of the intervention.
	Prabhakaran et al., mWellcare system with electronic decision support and SMS reminders.	To design and test a multifactorial electronic health record-and electronic decision support–based mHealth intervention across multiple sites at the primary care level using available trained staff.	mWellcare system was designed to generate electronic decision support (EDS) recommendations for the management of hypertension and diabetes mellitus, comorbid depression, and alcohol and tobacco use, tailored to the participant's profile and risk level. It stored the health records electronically, enabling long-term monitoring and follow-up. It was also equipped to send short message service reminders (to take medication and attend follow-up visits) to patients.	12 months	Enhanced Usual Care (EUC)_ Trained Physicians managed patients based on the charts displayed in clinics and clinical judgment, supported by nurses who provided lifestyle advice pamphlets in local languages. Follow-up care was determined at the discretion of the treating physicians.	No significant difference in the prespecified outcomes: SBP, HbA1c, Secondary outcomes: fasting blood glucose, total cholesterol, predicted 10-year risk of CVD, body mass index, depression, and tobacco and alcohol use.

	Schrader et al., eHealth management program using goACT platform to enable communication between patients and clinicians.	To provide a structured collaborative chronic disease management process addressing behaviours of both patient and clinician.	The intervention involved cognitive behavioural therapy, motivational interviewing, and behavioural psychotherapy, using tools like the Partners in Health scale (PIH), Cue and Response interview (C&R), and Problem and Goals assessment (P&G). Patients assessed their self-management skills using the PIH, and healthcare workers used the C&R in the goACT platform where results could be accessed and updated by patients and health care workers. The platform automated delivery of patient supports (such as action and appointment reminders), and email and SMS communication options to supplement any meetings or telephone contact agreed upon between patient and health care worker.	Not specified	Not available	Barrier: Burden of illness and low levels of information technology literacy hindered patient engagement.
