# Supplementary material for: Harnessing telehealth for multimorbidity management in rural and remote areas: A scoping review of interventions, outcomes, and implementation dynamics
Source: J Multimorb Comorb. 2025 Jun 11;15:26335565251344433. doi: 10.1177/26335565251344433 (PMC12163256; doi:10.1177/26335565251344433)
Supplement: Supplemental Material - Harnessing telehealth for multimorbidity management in rural and remote areas: A scoping review of interventions, outcomes, and implementation dynamics. [file sj-pdf-3-cob-10.1177_26335565251344433.pdf]

Supplemental File 1 Search strategy

Title of review: Harnessing telehealth for multimorbidity management in rural and remote areas: A scoping review of interventions, outcomes, and implementation dynamics.

Date Searched: 12 December 2024.

Database date coverage: 1 January 2010 – 12 December 2024

Other limits used: Language: English

**MEDLINE**		
(telehealth or "tele-health" or telemedicine or "tele-medicine" or telecare or "tele-care" or teleconsult* or "tele-consult*" or telemonitor* or "tele-monitor*" or ehealth* or "e-health*" or "electronic health*" or mhealth* or "m-health*" or "mobile health*" or "virtual health*" or "digital health*" or videoconferenc* or "video conferenc*" or "video consult*")<br>OR<br>(remot* adj2 (consult* or counsel* or monitor* or therap* or treatment* or delivery or sensing))	(comorbid* or "co-morbid*" or multimorbid* or "multi-morbid*" or "coexisting condition*" or "co-existing condition*")<br>OR<br>(multiple adj3 (diseases or illnesses or conditions or disorders or symptoms or diagnoses))<br>OR<br>(chronic adj2 (disease* or illness* or condition* or disorder* or symptom*))	(rural* or remote or regional* or village*)
**Subject headings (MeSH)**		
Telemedicine/ or Digital Health/ or Remote Consultation/ or Videoconferencing/ or Mobile Applications/ or Cell Phone/ or Smartphone/ or Internet/ or Computers, Handheld/ or Internet-based Intervention/	Comorbidity/ or Multimorbidity/ or Chronic Disease/ or Multiple Chronic Conditions/	Rural Health/ or Rural Population/ or Rural Health Services/

**PsycINFO**		
(telehealth or "tele-health" or telemedicine or "tele-medicine" or telecare or "tele-care" or teleconsult* or "tele-consult*" or telemonitor* or "tele-monitor*" or ehealth* or "e-health*" or "electronic health*" or mhealth* or "m-health*" or "mobile health*" or	(comorbid* or "co-morbid*" or multimorbid* or "multi-morbid*" or "coexisting condition*" or "co-existing condition*")<br>OR<br>(multiple adj3 (diseases or illnesses or conditions or disorders or symptoms or diagnoses))	(rural* or remote or regional* or village*)

"virtual health*" or "digital health*" or videoconferenc* or "video conferenc*" or "video consult*")<br>OR<br>(remot* adj2 (consult* or counsel* or monitor* or therap* or treatment* or delivery or sensing))	OR<br>(chronic adj2 (disease* or illness* or condition* or disorder* or symptom*))	
**Subject headings**		
Telemedicine/ or Teleconsultation/ or Videoconferencing/ or Mobile Applications/ or Mobile Health Applications/ or Mobile Health/ or Mobile Phones/ or Smartphones/ or Internet/ or Tablet Computers/ or Digital Interventions/	Comorbidity/ or Chronic Illness/	Rural Health/ or Rural Environments/

**Scopus (Elsevier)**		
(telehealth or "tele-health" or telemedicine or "tele-medicine" or telecare or "tele-care" or teleconsult* or "tele-consult*" or telemonitor* or "tele-monitor*" or ehealth* or "e-health*" or "electronic health*" or mhealth* or "m-health*" or "mobile health*" or "virtual health*" or "digital health*" or videoconferenc* or "video conferenc*" or "video consult*")<br>OR<br>(remot* W/2 (consult* or counsel* or monitor* or therap* or treatment* or delivery or sensing))	(comorbid* or "co-morbid*" or multimorbid* or "multi-morbid*" or "coexisting condition*" or "co-existing condition*")<br>OR<br>(multiple W/3 (diseases or illnesses or conditions or disorders or symptoms or diagnoses))<br>OR<br>(chronic W/2 (disease* or illness* or condition* or disorder* or symptom*))	(rural* or remote or regional* or village*)

**ProQuest Central**		
(telehealth or "tele-health" or telemedicine or "tele-medicine" or telecare or "tele-care" or teleconsult* or "tele-consult*" or telemonitor* or "tele-monitor*" or ehealth* or "e-health*" or "electronic health*" or mhealth* or "m-health*" or "mobile health*" or	(comorbid* or "co-morbid*" or multimorbid* or "multi-morbid*" or "coexisting condition*" or "co-existing condition*")<br>OR<br>(multiple NEAR/3 (diseases or illnesses or conditions or disorders or symptoms or diagnoses))	(rural* or remote or regional* or village*)

"virtual health*" or "digital health*" or videoconferenc* or "video conferenc*" or "video consult*")<br>OR<br>(remot* NEAR/2 (consult* or counsel* or monitor* or therap* or treatment* or delivery or sensing))	OR<br>(chronic NEAR/2 (disease* or illness* or condition* or disorder* or symptom*))	
